# Primary cutaneous melanoma of the breast skin: Incidence, characteristics, and management

**DOI:** 10.1016/j.jdin.2024.06.003

**Published:** 2024-07-24

**Authors:** Alexander S. Hines, Elliott H. Campbell, Jacob P. Reinhart, Olivia M. Crum, Sydney L. Proffer, Jerry D. Brewer, Lawrence E. Gibson, Addison M. Demer

**Affiliations:** Department of Dermatology, Mayo Clinic, Rochester, Minnesota

**Keywords:** breast melanoma, cutaneous melanoma of breast skin

*To the Editor:* Primary cutaneous melanoma arising in breast skin (hereafter referred to as “breast melanoma”) accounts for 0.28% to 3.8% of all cutaneous melanoma.^1^^,^^2^ Although the majority of existing data comes from older studies with more aggressive management strategies (ie, large surgical margins with more frequent axillary lymph node dissection and mastectomy), more recent data suggest that breast melanoma has a prognosis identical to melanoma elsewhere on the skin and should be managed the same.^1^, ^2^, ^3^ The purpose of this study was to characterize the incidence, pathologic features, management, and outcomes of breast melanoma. Following Institutional Review Board approval, using the Rochester Epidemiology Project (a multicenter medical records-linkage system encompassing residents of Olmsted County, Minnesota), we identified patients with a first lifetime diagnosis of breast melanoma between January 1, 1970, and December 31, 2020.

A total of 11 patients with breast melanoma were identified. Breast melanoma accounted for 0.48% (11/2310) of all primary cutaneous melanomas in our study, compared with 1.8% to 3.8% in prior studies.^2^^,^^4^ The overall incidence of breast melanoma was 0.29 per 100,000 patient-years. Patients were majority female (91%) with a median age at diagnosis of 40 years (range 20-73 years). In females, breast melanoma accounted for 0.92% (10/1091) of all primary cutaneous melanomas and incidence was 0.47 per 100,000 patient-years. Melanomas involved an upper breast quadrant (73%), lower breast quadrant (18%), or nipple/areola (9%). All cases were of the superficial spreading histologic subtype and median Breslow depth was 0.55 mm (range 0.34-1.1 mm).

All patients were treated with wide local excision (1 cm margins for all T1a/T1b melanomas; 1.5 cm for the sole T2a melanoma). Sentinel lymph node biopsy was performed in 2 cases (18%) and positive in 1 case. No patients received additional adjuvant or neoadjuvant treatments. There were no local recurrences, however 1 patient was diagnosed with widely metastatic melanoma 2 years after wide local excision. This patient had a positive sentinel lymph node biopsy with pathologic stage of IIIa (T2a, N1a, M0) at the time of melanoma excision (only a single metastatic cell was identified on the sentinel lymph node biopsy and the patient opted for monitoring). Median follow-up duration was 8.8 years and no deaths were recorded. Our study confirms previous findings that breast melanomas are most commonly of the superficial spreading subtype and more common in the upper breast quadrants (which has been suggested to be related to higher sun exposure in this location).^2^^,^^3^^,^^5^

The main limitation of this study include is its retrospective nature and small sample size. Additionally, it is possible that some breast melanomas may have been missed if the tumor was labeled as another anatomic site (ie, chest). This seems more likely to occur in male patients where the breast tissue is often less pronounced, possibly explaining the high female to male ratio noted in our study. Additional research with larger samples is necessary to support the current recommendation that breast melanomas be managed identically to melanomas elsewhere on the skin.

## Conflicts of interest

None disclosed.
